# Simulating gene-gene and gene-environment interactions in complex diseases: Gene-Environment iNteraction Simulator 2

**DOI:** 10.1186/1471-2105-13-132

**Published:** 2012-06-14

**Authors:** Michele Pinelli, Giovanni Scala, Roberto Amato, Sergio Cocozza, Gennaro Miele

**Affiliations:** 1 Gruppo Interdipartimentale di Bioinformatica e Biologia Computazionale, Università di Napoli “Federico II” - Università di Salerno, Italy; 2 Dipartimento di Biologia e Patologia Cellulare e Molecolare “L. Califano”, Università di Napoli “Federico II”, Napoli, Italy; 3 Dipartimento di Scienze Fisiche, Università di Napoli “Federico II”, Complesso Universitario di Monte S.Angelo, Napoli, Italy; 4 INFN Sezione di Napoli, Napoli, Italy

**Keywords:** Gene-environment interaction, Computer simulation, Complex disease, Epistasis, Genetic, Genome-wide association study, Genetics, Population, SimuPOP, Linkage disequilibrium, Genomics

## Abstract

**Background:**

The analysis of complex diseases is an important problem in human genetics. Because multifactoriality is expected to play a pivotal role, many studies are currently focused on collecting information on the genetic and environmental factors that potentially influence these diseases. However, there is still a lack of efficient and thoroughly tested statistical models that can be used to identify implicated features and their interactions. Simulations using large biologically realistic data sets with known gene-gene and gene-environment interactions that influence the risk of a complex disease are a convenient and useful way to assess the performance of statistical methods.

**Results:**

The Gene-Environment iNteraction Simulator 2 (GENS2) simulates interactions among two genetic and one environmental factor and also allows for epistatic interactions. GENS2 is based on data with realistic patterns of linkage disequilibrium, and imposes no limitations either on the number of individuals to be simulated or on number of non-predisposing genetic/environmental factors to be considered. The GENS2 tool is able to simulate gene-environment and gene-gene interactions. To make the Simulator more intuitive, the input parameters are expressed as standard epidemiological quantities. GENS2 is written in Python language and takes advantage of operators and modules provided by the simuPOP simulation environment. It can be used through a graphical or a command-line interface and is freely available from http://sourceforge.net/projects/gensim. The software is released under the GNU General Public License version 3.0.

**Conclusions:**

Data produced by GENS2 can be used as a benchmark for evaluating statistical tools designed for the identification of gene-gene and gene-environment interactions.

## Background

Most of the common human diseases with high mortality rates (such as cancer, heart disease, obesity, diabetes, and several common psychiatric and neurological conditions) are classified as complex diseases [1,2]. By definition, a complex disease is a multifactorial complex trait generally caused by multiple predisposing loci (possibly interacting) and by the exposure to particular environmental factors [3]. Although several genetic and environmental factors have been shown to affect susceptibility to particular complex diseases, the intricate sets of relationships between these factors and disease susceptibility are not yet exhaustively understood. For this reason, typically, the proportion of risk accountable to genetics and environment remains mostly unpredictable [4]. Explanations for their unpredictability might include the occurrence of still unidentified factors and/or the presence of non-linear interactions among already identified factors; for example, some combinations of genetic and environmental factors could have disease risks that are consistently higher than those predicted by a single component.

Gene-environment interactions (G×E) are expected to influence complex phenotypes, for example, disease risk. Hence individuals with predisposing genetics are more likely to develop a disease when exposed to a damaging environment than individuals, exposed to the same environment, without predisposing genetics [5,6]. The role of G×E is so relevant that it is generally accepted that neglecting them can lead to an underestimation of disease risk, and may explain some of the inconsistencies in replications in different studies [7].

Complex phenotypes are regulated by pathways and biochemical mechanisms that involve many genetic products. Hence, in addition to interactions among genes and environment, interactions among different genetic loci (G×G) can also influence disease risk. In particular, G×G are defined as epistatic when the allelic variations of one gene alters the effect of variations of another gene [8]. Epistasis has been identified in human diseases [9,10], and its role in public health has been highlighted [8].

Surprisingly, despite the general agreement on the relevance of G×E and G×G for correct disease risk estimations, only a few epidemiological studies have attempted to identify them. Indeed, studying the complex interactions among risk factors is a daunting task that requires large data sets and specific research designs. Furthermore, the best statistical method for the identification of G×G and G×E in case-control data sets [11,12] is still widely debated. The performance of statistical methods that are used for the identification of G×G and G×E are typically influenced by many factors: sample size, number of involved factors, type of interaction, model of inheritance, allelic frequencies, distributions of the environmental factors, and relative strength of different factors affecting disease risk. Unfortunately, only a few of these features are generally assessable in real populations. A further limitation of the epidemiological studies that have been performed so far, is the limited knowledge about the impact of linkage disequilibrium (LD) on association statistics in the presence of G×G and G×E. It has been demonstrated that when G×G occur and the assayed SNPs are not the functional ones but SNPs that are in LD with them, common statistics like *r*^2^ are generally inappropriate and tend to lead to an over/underestimation of disease risk [13].

A possible strategy to assess the performances of statistical methods is to test them against simulated data sets where the relevant features influencing the disease risk are known (for a review of genetic simulators see [14] and the North Shore LIJ Research Institute List of Genetic Analysis Software [15]). With this aim, some of the authors [14] of the present work proposed a novel approach to simulate case-control samples based on: 

1. a Multi-Logistic Model (MLM) that can model any type of G×G and G×E,

2. a mathematical approach (Knowledge Aided Parameterization System, KAPS) that can translate biological and epidemiological information to MLM parameters, and

3. GENS (Gene Environment iNteraction Simulator), a software that produces simulated data sets.

Using that approach interactions between one genetic and one environmental factor only could be simulated; therefore, it was not possible to account for epistatic G×G. Moreover, all simulated loci were considered to be independent and thus it was not possible to account for LD [16].

In the present paper, we describe an extension of the previous model that overcomes such limitations using a new strategy that simulates up to two-genes×one-environment interactions with the possible inclusion of epistasis. Importantly, the present algorithm can be easily extended to manage more than two genetic and one environmental factors. However, to simplify the design of biologically meaningful interactions, we limited the number of features (see the Discussion section for details). Furthermore, the inclusion of two genetic factors (with epistatic interaction) that in turn interact with a continuous environmental factor heavily increased of the complexity of the model. Indeed, statistical methods that can deal with even two genetic factors are still far from being functionally useful for real, large data sets [17]. To provide a realistic genetic background to the simulated populations, we implemented our extended model as a module which can be integrated with simuPOP, a forward-time populations simulator that reproduces realistic demographic and evolutionary features [18].

## Implementation

### GENS2 workflow

**Figure 1:**
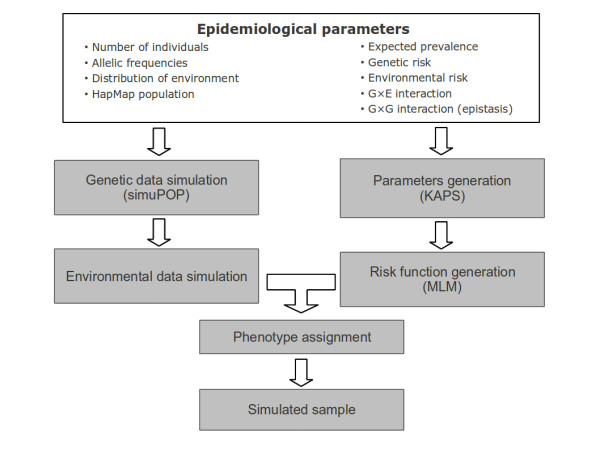
**GENS2 work flow.** Chart of the steps that were used to simulate a complex disease in a population using the simuPOP and GENS2 systems.

Figure 1 depicts the GENS2 algorithm flowchart that is used to generate case/control synthetic populations starting from a set of desired epidemiological parameters (Table 1). The simulation procedure has two main branches: the definition of genetic and environmental information for each individual (left side of Figure 1), and the translation of user desired epidemiological parameters along with G×E and G×G models into the corresponding MLM parameters (right side of Figure 1). The two branches merge in the last step of the procedure, where disease status is assigned to each individual. In the following sections we will describe the three parts of the algorithm in detail, emphasizing the main advances of the new software over the previous one.

**Table 1:** The epidemiologic parameters that were used for the sample simulation

**Task**	**Required parameters**	**Description**
**SimuPOP**		
1) Starting data (Hap Map)	Chromosomes, or chromosome regions, or markers and marker distance.	The genomic regions containing the loci that will be simulated
	Population (ethnicity)	The starting frequency and linkage data to be used in the sumulation
2) Simulation of sample’s genetic data	DPLs (Disease Predisposing Loci)	Loci that will influence the disease risk.
	Target allelic frequency	Final allelic frequencies at the end of simuPOP simulation
	Final sample size	Number of individuals that compose the population by simuPOP
**GENS2**		
Starting sample	simuPOP generated sample	Sample data generated with simuPOP
	Disease prevalence	The expected disease prevalence in the whole sample
Environment	Environmental factor distribution	Distribution of the environmental exposure in the whole sample
	Environmental factor OR	Odds ratio associated with one-unit-increase of the environmental exposure
	Noisy Environmental variables	As many as desired confounding environmental exposures not associated with the disease risk (gaussian, binomial or uniform distributed)
Genetics	DPLs	These are the same DPLs as selected in the simuPOP simulation
	High risk alleles	The allele, for each DPL, associated with the highest disease risk
	DPLs genotypic RR	The relative risk of the high risk homozygote versus low risk homozygote, for each DPL
	Dominance	The relationship of the risk associated with the heterozygote with that associated with the homozygotes (recessive, dominant, codominant)
	Epistasis model (G×G)	Percent increase of the risk associated with each combined genotype
Gene Environment interaction	G×E model	One of the four predefined interaction models

#### Generation of the synthetic data set

The generation of the starting sample is carried out by a series of simuPOP scripts [19] that 

• download phased genomic data from the HapMap public database [20],

• select a subset of SNPs or entire genomic regions, and

• let the population evolve until it reaches the desired size and frequencies for some disease predisposing loci (DPLs).

To obtain a synthetic data set, simuPOP drives a forward-time simulation to obtain a population that is composed of the desired number of individuals and genotypic frequencies for all the markers. The use of this simulator helps to retain genetic realism in the final population, in particular with respect to the patterns of LD (for a detailed description of this process, please see [19]). When the genetic information for each individual has been obtained, GENS2 assigns environmental exposures following a user defined Gaussian distribution for the disease environmental variable, and several other user defined distributions (Gaussian, Uniform or Binomial) for a number of other environmental variables not related with the disease (environmental-confusing variables).

#### Definition of the penetrance model

The second branch of the simulation procedure (right side of Figure 1) is implemented in the Knowledge Aided Parameterization System 2 (KAPS2) subsystem which accepts the input of some standard epidemiological measures for the relevant features listed here: 

• the expected prevalence of the disease in the sample,

• the *id* in the input data set of one or two DPLs,

• the allelic frequencies of DPLs (calculated automatically from the input population),

• the effect on disease risk of each DPL in terms of the relative risk of the high risk homozygote compared with the other homozygote,

• the dominance relation of each DPL (W), expressed as a number in the interval [0−1], representing the dominance relation (W=0 dominant, W=1 recessive, 0<*W*<1 co-dominant), and

• the distribution parameters and the effect of the environmental factor on disease risk, expressed as odds ratio (OR) of the risk related to one-unit increase in the exposure.

KAPS2 also requires G×E and G×G models when two DPLs are provided. In particular: 

i) G×E models are chosen from a set of four predefined models, two models of interaction between DPLs and the environment, and two special models in which there is no gene-environment interaction but in which only one genetic or environmental factor contributes to the disease risk (see Table 2).

**Table 2:** Predefined gene-environment interaction models in GENS2

**Interaction model**	**Description**
Genetic Model(GEN)	Disease risk depends only on the genetics of an individual
Environmental Model (ENV)	Disease risk depends only on environmental exposure of an individual
Gene Environment interaction Model (GEM)	The genetics modifies the effect of the environment in modulating the disease risk
Additive Model(ADD)	The effects of environment and genetics are independent and sums in modulating the disease risk

ii) G×G models (epistasis) are accepted in the form of percentage variations on the risk associated with a maximum number of three (out of the possible nine) combined genotypes.

**Figure 2:**
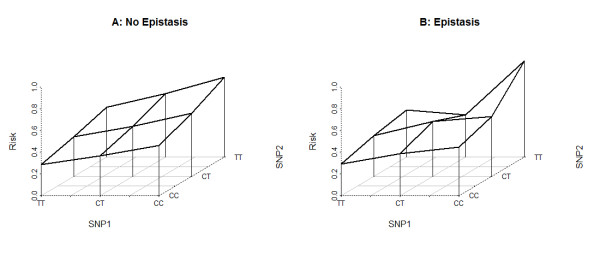
**Example of application of epistasis.** Disease penetrance for combined genotypes before (left panel) and after (right panel) the application of an epistasis model with an increment of 20% of the risk associated with the (CC-TT) composed genotype. The x- and y- axes plot the reported genotypes of the two DPLs; the z-axis plots the risk associated with each combined genotype.

KAPS2 converts population features and G×E and G×G models into the corresponding parameters of the MLM in two steps. First, starting from the provided epidemiological parameters, KAPS2 calculates the penetrance of each combined genotype assuming no interaction between the genotypes of each locus. Epistasis (if defined) is then modeled through a deformation procedure, reflecting G×G variations, of the set of penetrance values that keeps it coherent with user defined epidemiological parameters. In this step, when there is more than one way to change the values of the set (i.e. less than three epistatic variations are provided), a mathematical optimization system is employed to find the deformation characterized by the smallest variation on the values not constrained by user defined epistatic variations. An example of the results of the epistasis application is presented in Figure 2. In particular, the figure shows the disease penetrance for each combined genotype before (left panel) and after (right panel) the penetrance of one combined genotype (namely (3,3)) has increased by 20%. Thus, by following the procedure presented above, the variations in the disease penetrance values of other combined genotypes are automatically distributed.

Consequently, for each combined genotype, KAPS2 computes the coefficients of a penetrance function of the environmental exposure that is associated with the combined genotype in the MLM. In this step G×E are also modeled; the Additive model (ADD) assumes that combined genotypes with higher penetrance have a higher basal disease risk, while the risk associated with the environmental factor is just added. On the other hand, in the modulative model (GEM), combined genotypes with higher penetrance have the same basal risk although they are more ’sensitive’ to the effect of the environment (see the Methods section).

#### Disease risk of an individual

In the final step the two branches of the procedure (Figure 1) join. Once the genetics and the environmental exposure for each individual are given, its disease risk can be obtained by evaluating the penetrance function associated with its combined genotype. The risk is then used to assign a disease status using a random process.

### Software

To create simulated populations, we employed an existing tool, SimuPOP, and the implementation of the above described algorithm. Using SimuPOP it is possible to drive a forward-time simulation that results in a population composed by the desired number of individuals and having specified genotype frequencies for a set of markers. To be usable in GENS2, populations should be created in SimuPOP as described previously [19]. This procedure generates a data set that contains genotypic data as a set of bi-allelic loci, for each individual in the population.

GENS2 accepts as input a population produced by SimuPOP and the *ids* of the DPLs chosen from those present in the data set. For each DPL, the penetrance values are provided as relative risk (RR) and dominance model (see [14]). RR is calculated as the risk ratio of the high over the low risk homozygotes. For each combination of DPL genotypes, the percentage variation of the penetrance value if epistasis occurs can be assigned. GENS2 assigns environmental factor exposure on the basis of a user-defined Gaussian distribution. Any remaining non-implicated loci can be considered as background noise. Similarly, GENS2 can produce an arbitrary number of non-associated random environmental exposures, following a Gaussian, uniform or binomial distribution, that act as further background noise.

On the basis of the selected type of G×G and G×E, GENS2 calculates the coefficients of the MLM as described in the Method section.

For each individual, GENS2 assigns the disease status (affected or unaffected) on the basis of its disease risk by applying the MLM and using a random process.

The main output of the software can be either a single file or several files for a set of subpopulations of a given size produced by means of a subsampling procedure. Subsampling allows bootstrapping procedures to be executed on data sets produced with the same features. The output of GENS2 is in the form of a table in which each row represents an individual and the columns contain, from the left to the right, disease status, gender, environmental exposures and genotypes for each individual.

Two possible formats for the genetics output are available: phased haplotypes or genotypes. In both output formats the initial columns are identical to those described above; however, they differ in the way the genetic information for each individual is represented. In the phased haplotype format, there are two columns for each SNP that report the allele status (either A, C, T or G) on each chromosome. In the genotype format, each SNP is represented by one number (1, 2 or 3), where 2 represents the heterozygote and 3 represents, for DLPs, the high risk homozygote or, for all the other SNPs, the lower frequency homozygote.

In addition to the main output file, GENS2 outputs a log file that contains an extensive report of all the intermediate steps in the procedure and the values used to obtain the populations. Optionally, a file containing the *ID* and genomic position of the SNPs in the data set can be returned.

GENS2 is designed to be used either from the command line as a Python script, or through a graphical user interface, similar to a wizard, that prompts the user in the specification of all required parameters [see Additional file 1].

Overall, the computational time complexity of the simulation procedure depends by both simuPOP and GENS2. For GENS2, the procedure is dominated by the assignment of the disease status to all individuals in the population. Indeed, after the KAPS2 module has performed the translation of user provided parameters into MLM parameters in bounded constant time, the time complexity becomes linear in the number of individuals and the number of represented variables (genotypes and environmental exposures) for each individual in the simulated population. On the other hand, the amount of time required to perform a simulation with simuPOP depends on the size of the simulation and scales roughly linearly with the number of markers and individuals that are used [19]. GENS2 is written in Python and is completely open-source. The software is freely available from [21].

## Results and discussion

Here we describe a method based on the MLM to simulate two genetic and one environmental factors interacting in the determination of a disease risk. The method is implemented in GENS2, a software that is freely available.

To test populations produced by GENS2, we performed a set of analyses on some representative populations. The aim was to emulate a case in which GENS2 was used to assess the performances of a feature selection method. In particular, all the analyses were performed using a logistic regression (*glm* function in R) with a different model for each test and considering the status as the dependent variable.

The first test was a single-marker analysis on a population of 1,000 cases and 1,000 controls with two DPLs in two distinct genomic regions, with no epistasis and an additive G×E model. The association of each marker with the status was tested using logistic regression analyses with model: disease risk = genetic factor + environmental factor. As expected, the most significant associations were those of DPLs [see Additional file 2]. The result showed that the environmental variable was also associated with the disease (*p*<10^−6^). Furthermore, non-causative markers in LD with the two DPLs also showed a significant association that was roughly proportional to the value of *r*^2^ with the DPLs.

The second test was similar to the first, except that 10,000 cases and 10,000 controls and a modulative G×E model for the DPLs were used. For this test, the logistic regression was used by considering both an additive model (disease risk = genetic factor + environmental factor) and a multiplicative model (disease risk = genetic factor * environmental factor). None of the markers, when tested by additive model, reached a Bonferroni corrected significance level [see Additional file 3, middle panel]. Conversely, DPLs were found to be significant when the multiplicative model was explicitly considered. Non-causative markers in LD with them were also found to be significant. Notably, this more complex model required a 10-fold increase in the sample size to achieve the same significance level as the previous test.

**Figure 3:**
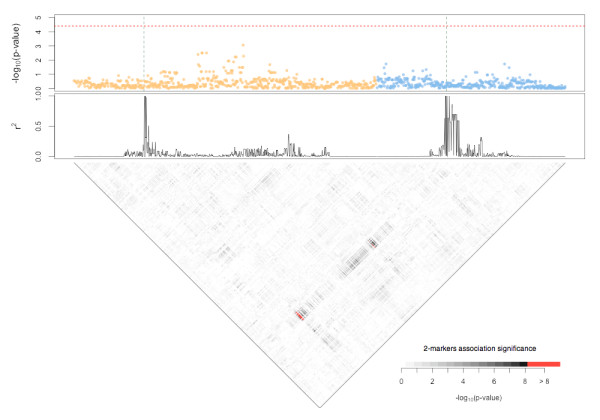
**Association test for the case of epistatic interaction.** The population comprised 5,000 cases and 5,000 controls. Two DPLs with no marginal risk (RR=1), an epistatic interaction ( + 20*%*penetrance for the (3,3) combined genotype) and an additive G×E model (odds ratio (OR)=1.2) were used. The two DPLs are in two distinct genomic regions (Chr 8: 117,948,182 - 119,256,695 in yellow; Chr 10: 114,408,939 - 115,256,799 in cyan). In the upper panel, the Manhattan plot shows the significance of the association (−log_10_(p-value)) of each marker when tested individually (each dot represents a different marker). The red dashed line represents the significance threshold (0.05 after Bonferroni correction) and the green dashed lines mark the position of DPLs. In the middle panel, the *r*^2^ of each marker with the DPL in the same region is shown. In the bottom panel, the significance of the association for each 2-loci interaction (grey scale, nonsignificant; red scale, significant at a 0.05 level after Bonferroni correction) is shown.

Finally, we tested an example of two DPLs with no marginal risk, an epistatic interaction ( + 20*%* penetrance for the (3,3) combined genotype) and an additive G×E model in a population of 5,000 cases and 5,000 controls. Because of the higher computational cost of this analysis, we performed the test on only a subset of about 1,200 markers surrounding the two DPLs. The results are displayed in Figure 3. The top panel shows the results of a single-marker analysis. As expected, no markers were found to be significantly associated when tested individually. Thus, all possible 2-markers interactions (bottom panel) were tested. Only the gene-gene interactions of DPLs and of markers in strong LD with them were found to be significantly associated with the status after a Bonferroni correction (red dots).

The model described here can handle, in principle, any number of DPLs and environmental variables. However, we chose to limit the implementation to a relatively small number of factors (two genetic and one environmental) so that setting up the model does not become too complicated for the user. In this way, we reached a balance between the complexity of the represented phenomena and simplicity in the definition of the model. Moreover, the best strategy to identify even simple interactions as single G×G and G×E with binary environmental variables it is still debated (for an example of the debate, see the report on the 2009 Genetic Analysis Workshop [11,12,17]). For this reason, we believe that a set of simulated populations in which all features are known provides an important tool for the identification of the best strategy to identify and study G×G and G×E.

Several methods simulating genetic data have been proposed, many of them also handle complex LD patterns and polygenic traits [22,23]. For example, HAPGEN2 [22] simulates multiple polymorphic loci that are in LD and in the same chromosomal region. HAPGEN2 can simulate G×G, including epistasis, between two loci; however, the available models are limited to a predefined set. Moreover, this program cannot handle G×E and the number of cases and controls that are produced cannot be controlled. Another tool, *gs*[23], similarly simulates multiple loci with a realistic pattern of LD; it can modulate a quantitative trait (as disease risk) and can also manage G×G and G×E. In *gs* the user can define G×G between two loci in two ways: one, by providing a penetrance matrix for combined genotypes or, two, by selecting a penetrance matrix from a predefined set of G×G models. However, both these approaches have some limitations. Although the first allows great control over the penetrance of each genotype, it easily leads to a loosening of control on marginal effects, making the replication of real populations difficult. The second approach, on the contrary, is too restrictive and does not allow any possible interaction to be simulated. For simulation of G×G, *gs* allows the user to input a list of rules regarding specific combinations of genotypes and levels of environmental values, and the corresponding risk levels. Again, this approach makes it very difficult to control the overall characteristics of populations in terms of marginal effects of genetic and environmental factors. The approaches described above can simulate complex interactions by loosening control on overall population characteristics or, alternatively, can keep the overall population characteristics under control by limiting the freedom to specify the interactions. Another strategy to simulate G×G and G×E is to manually write functions or sets of rules that associate each combination of genes and environmental factors to a risk value. Although this strategy provides more freedom, it is very difficult to set up when the control of marginal effects of single factors is desired. We believe that GENS2 provides a better balance between the freedom to define possible interactions among factors and the control of the overall population characteristics.

The simulated populations produced with GENS2 can be thought of as a sampling of an ideal infinite population that has the characteristics specified by the user. From this point of view, it is easy to understand that fluctuations of observed values around the expected ones can occur. Among the elements that mostly affect these fluctuations, are sample size, allele frequencies, and penetrance values. In particular, small sample sizes increase the effect of sampling error and thus, as expected, these fluctuations tend to vanish as the sample size is increased [see Additional file 4]. This property can be used to test statistical methods because it allows the user to assess how well the methods perform as population and sample features change; conversely, it helps to assess the statistical power of methods in the presence of population features that can only be presumed.

Although the GENS2 part of the simulation process is reasonably fast, the procedure to simulate large populations using simuPOP takes time to complete. It would be difficult to simulate a large number of samples without a cluster system, unless multiple (small) samples are drawn from the same large population.

## Conclusions

GENS2 allows the simulation of gene-gene and gene-environment interactions among two genetic and one environmental factor in relation to the risk to develop a complex disease. It is based on data with a realistic pattern of LD and it has no limitations either on the number of individuals that can be simulated or on the number of genetic and environmental factors within a simulated data set. Furthermore, a large amount of effort has been channeled into allowing the input of parameters as standard epidemiological quantities so that the software is immediately usable by the biomedical community.

GENS2 provides large biologically realistic data sets with known features that can be used to challenge, and eventually improve, the statistical tools that are designed to identify those interactions.

## Methods

Here we present the mathematical background underlying the extension of the earlier model [14] to the case of two (possibly interacting) DPLs. For simplicity, we have described the case of two DPLs and one environmental factor. Thus, we can generally assume that: 

1. the genetics can influence the disease risk either directly or by modifying the effect of the environment.

2. the genetic loci can have independent effects (no epistasis) or can interact in an epistatic manner, and

3. the DPLs are not in LD.

### The Multi-Logistic Model

To model these situations we applied the MLM, here briefly summarized, that uses a different logistic function for each combination of the two genotypes [14]. The dependent variable of the functions is the disease risk while the independent variable is the environmental exposure. For diploid loci, denoted by A and B two disease predisposing alleles, there are three distinct genotypes for each locus, namely AA, Aa, aa and BB, Bb, bb. For an individual carrying a combined genotype (*g*_*a*_*g*_*b*_) (with *g*_*a*_∈*GA*={*AAAaaa*} and *g*_*b*_∈*GB*={*BBBbbb*}) who is exposed to the environmental level *x*, the disease risk is defined under the MLM by the conditional probability *P*(affected|*g*_*a*_*g*_*b*_*x*), which is parameterized as:

(1) $$P\left(\text{affected}\left|g_{a},g_{b},x\right)\right)\;=\;{\left[1\;+\;\exp\;\left\{\alpha_{\left(g_{a},g_{b}\right)}+\beta_{\left(g_{a},g_{b}\right)}\;x\right\}\right]}^{-1}$$

where $\alpha_{\left(g_{a},g_{b}\right)}$ and $\beta_{\left(g_{a},g_{b}\right)}$ are free parameters determined by the genetic factors that determine the shape of the function.

To simulate a population, the coefficients $\alpha_{\left(g_{a},g_{b}\right)}$ and $\beta_{\left(g_{a},g_{b}\right)}$ of the logistic functions that produce the desired population features have to be determined. This task is performed by the KAPS2 module that finds the corresponding MLM coefficients by considering all the desired population features within a set of biological constraints.

### Determination of MLM parameters

Let $P_{g_{a}}$ and $P_{g_{b}}$ be the genotypic frequencies for variables A and B and let *m* be the prevalence of the disease. Starting from these values and using the independence hypothesis for the variables A and B, the probability $P_{\left(g_{a},g_{b}\right)}$ for an individual to carry the genotype (*g*_*a*_,*g*_*b*_) is the product $P_{g_{a}}\;P_{g_{b}}$. If for each combined genotype (*g*_*a*_,*g*_*b*_) the total risk for the disease insurgence $TR_{\left(g_{a},g_{b}\right)}$ is known, then this parameter represents the probability for an individual to be affected given the carried genotype (*g*_*a*_,*g*_*b*_). The value of this parameter is obtained with the MLM as

(2) $$\begin{array}{lll} P(\text{affected}|g_{a},g_{b})\; & =\;\underset{X}{\int }\;\frac{P^{E}(x)}{\left[1\;+\;\exp\;\left\{\alpha_{\left(g_{a},g_{b}\right)}\;+\;\beta_{\left(g_{a},g_{b}\right)}\;x\right\}\right]}\;dx\; & \;\\ & \equiv TR_{\left(g_{a},g_{b}\right)}\; \end{array}$$

where *X* is the domain of the environmental variable.

Because every logistic function in MLM is characterized by its own parameters, the 3×3 pairs of values ($\alpha_{\left(g_{a},g_{b}\right)},\beta_{\left(g_{a},g_{b}\right)})$ that satisfy the constraints expressed by equation (2) need to be found.

#### Modeling G×E

In general, equation (2) admits infinite solutions. However, the G×E model imposes some constraints on the coefficients. Thus, by fixing the value of one of the coefficients $\beta_{\left(g_{a},g_{b}\right)}$, hereafter denoted as *β*_*AB*_, the number of degrees of freedom of the system can be reduced, drawing one solution from the equation system. By convention, we chose to associate *β*_*AB*_to the genotype with highest risk; it is easy to show that this value corresponds to the natural logarithm of the odds ratio of the risk which is related to the increase of one unit of the environmental exposure. Constraints imposed on the system by each one of the proposed gene environment interactions model are summarized below: 

• Genetic effect (GEN): $\alpha_{\left(g_{a},g_{b}\right)}\neq 0$ and $\beta_{\left(g_{a},g_{b}\right)}=0\forall \left(g_{a},g_{b}\right)\in \mathrm{GA}\times \mathrm{GB}$ and $Â\neg [\alpha_{\left(g_{a},g_{b}\right)}=\alpha_{\left(g_{x},g_{y}\right)}\forall \left(g_{a},g_{b}\right),\left(g_{x},g_{y}\right)\in \mathrm{GA}\times \mathrm{GB}]$.

• Environmental effect (ENV): $\alpha_{\left(g_{a},g_{b}\right)}=\alpha_{\left(g_{l},g_{k}\right)}$ and $\beta_{\left(g_{a},g_{b}\right)}=\beta_{\left(g_{l},g_{k}\right)}=\beta_{\mathrm{AB}}\neq 0\forall \left(g_{a},g_{b}\right),\left(g_{l},g_{k}\right)\in \mathrm{GA}\times \mathrm{GB}$.

• Modulative effect (GEM): $\alpha_{\left(g_{a},g_{b}\right)}=\alpha_{\left(g_{l},g_{k}\right)}$ and $\beta_{\left(g_{a},g_{b}\right)}\neq 0\;\forall \left(g_{a},g_{b}\right),\left(g_{l},g_{k}\right)\in \mathrm{GA}\times \mathrm{GB}$.

• Additive effect (ADD): $\alpha_{\left(g_{a},g_{b}\right)}\neq 0$ and $\beta_{\left(g_{a},g_{b}\right)}=\beta_{\mathrm{AB}}\neq 0\;\forall \left(g_{a},g_{b}\right)\in \mathrm{GA}\times \mathrm{GB}$.

When the interaction model, the matrix containing total risk values for each combination of genotypes, namely $TR_{\left(g_{a},g_{b}\right)}$, and the value for the coefficient *β*_*AB*_have been defined, a set of six transcendent equations can be written with the coefficients of the logistic functions (except *β*_*AB*_) as the unknown variables; these equations admit exactly one solution [14].

#### Modeling G×G

To determine $TR_{\left(g_{a},g_{b}\right)}$, further assumptions concerning the role played by G×G are required. First, the values that are used need to be consistent with the provided disease prevalence *m*, namely

(3) $$P(\text{affected})=\underset{g_{a}\in \mathrm{GA}}{\sum }\underset{g_{b}\in \mathrm{GB}}{\sum }TR_{\left(g_{a},g_{b}\right)}P_{\left(g_{a},g_{b}\right)}\equiv m$$

Moreover, the total risk values associated with the genotypes of a single locus are related to those of combined genotypes via marginalization:

(4) $$P(\text{affected}|g_{a})=\frac{1}{P_{g_{a}}}\underset{g_{b}\in \mathrm{GB}}{\sum }TR_{\left(g_{a},g_{b}\right)}P_{\left(g_{a},g_{b}\right)}\equiv TR_{g_{a}}$$

(5) $$P(\text{affected}|g_{b})=\frac{1}{P_{g_{b}}}\underset{g_{a}\in \mathrm{GA}}{\sum }TR_{\left(g_{a},g_{b}\right)}P_{\left(g_{a},g_{b}\right)}\equiv TR_{g_{b}}$$

In general, once the marginals $TR_{g_{a}}$ and $TR_{g_{b}}$ are given, there are infinite choices for the matrix $TR_{\left(g_{a},g_{b}\right)}$ that satisfy the constraints imposed by Eq.s (4) and (5); each matrix is representative of a particular G×G model. Of the possible choices, the case of no epistasis represents a situation where $TR_{\left(g_{a},g_{b}\right)}$ is determined starting from the fixed values of $TR_{g_{a}}$ and $TR_{g_{b}}$ only. In such a model, genetic factors independently contribute to the probability of being affected and are conditionally independent given the disease status. Under these assumptions the following relationship which satisfies the constraints of Eq.s (4) and (5) is easily obtained:

$$TR_{\left(g_{a},g_{b}\right)}^{I}=\frac{TR_{g_{a}}\;TR_{g_{b}}}{m}$$

Notice that the superscript “I” is a reminder that the independent polygenic model has been assumed.

Using an independent polygenic model and a deformation procedure, epistatic interactions among DPLs can be modeled to obtain a matrix $TR_{\left(g_{a},g_{b}\right)}^{E}$ (where superscript “E” stands for epistatic) that still complies with constraints (4) and (5). In this approach, epistasis is modeled as a departure from the independent polygenic model via a change (positive or negative) in one or more entries of $TR_{\left(g_{a},g_{b}\right)}^{I}$.

Let $\Delta \in R_{\left[-1,1\right]}^{3\times 3}$ be a matrix with the same dimensionality as *TR*^*I*^, where each entry $\Delta_{\left(g_{a},g_{b}\right)}$ represents the variation of the element $TR_{\left(g_{a},g_{b}\right)}^{I}$ as a result of the epistatic interaction of the combined genotypes (*g*_*a*_,*g*_*b*_). By definition $TR_{\left(g_{a},g_{b}\right)}^{E}=TR_{\left(g_{a},g_{b}\right)}^{I}+\Delta_{\left(g_{a},g_{b}\right)}$ and must satisfy the condition

(6) $$0\leq TR_{\left(g_{a},g_{b}\right)}^{E}\leq 1\;\;\;\;\forall \left(g_{a},g_{b}\right)$$

Using the expressions in Eq.s (4) and ( 5) we get

(7) $$\begin{array}{ll} \underset{g_{b}\in \mathrm{GB}}{\sum }(TR_{\left(g_{a},g_{b}\right)}^{I}+\Delta_{\left(g_{a},g_{b}\right)})P_{g_{b}}\;\;=\;\;TR_{g_{a}} & \; \end{array}$$

(8) $$\begin{array}{ll} \underset{g_{a}\in \mathrm{GA}}{\sum }(TR_{\left(g_{a},g_{b}\right)}^{I}+\Delta_{\left(g_{a},g_{b}\right)})P_{g_{a}}\;\;=\;\;TR_{g_{b}} & \; \end{array}$$

Because by construction, the matrix $TR_{\left(g_{a},g_{b}\right)}^{I}$ already satisfies the constraints (4) and (5), the two following consistence conditions can be derived,

(9) $$\begin{array}{ll} \underset{g_{b}\in \mathrm{GB}}{\sum }\Delta_{\left(g_{a},g_{b}\right)}P_{g_{b}}\;\;=\;\;0 & \; \end{array}$$

(10) $$\begin{array}{ll} \underset{g_{a}\in \mathrm{GA}}{\sum }\Delta_{\left(g_{a},g_{b}\right)}P_{g_{a}}\;\;=\;\;0 & \; \end{array}$$

Once the quantities $TR_{g_{a}}$, $TR_{g_{b}}$, $P_{g_{a}}$ and $P_{g_{b}}$ are given, the constraints (9) and (10) define a convex region in **R**^3×3^ in which the elements are assignments for the entries of matrix $\Delta_{\left(g_{a},g_{b}\right)}$. The specification of an epistatic model is, therefore, made through the definition of an increments matrix $\Delta_{\left(g_{a},g_{b}\right)}$ that complies with the constraints (9) and (10) and that also satisfies the positivity condition for $TR_{\left(g_{a},g_{b}\right)}^{E}$. It can be difficult for a user to specify such a matrix in a way that does not violate the above constraints. However, the number of entries of $\Delta_{\left(g_{a},g_{b}\right)}$ that the user has to provide (paying attention to avoiding extreme or off-range values) can be reduced by letting the system find the remaining entries.

More precisely (in the two variables case), given the constraints of Eq.s (9) and (10) from one up to three entries for $\Delta_{\left(g_{a},g_{b}\right)}$ can be provided following the rule that any pair must lie on the same row or in the same column. If the user correctly provides three values, the system admits only one assignment for unspecified values of $\Delta_{\left(g_{a},g_{b}\right)}$; however, if the user provides less than three values, there are an infinite number of ways to choose the remaining entries of $\Delta_{\left(g_{a},g_{b}\right)}$. In such a case, instead of randomly choosing a solution, a solution that maximizes an “objective function” is chosen. The problem of fixing the remaining values of $\Delta_{\left(g_{a},g_{b}\right)}$ can be represented as a continuous mathematical programming problem with decision variables that are the non-user-provided entries of $\Delta_{\left(g_{a},g_{b}\right)}$ and whose admissible region can be determined by Eq.s (9), (10) and (6).

An objective function can be used to minimize the variance of the set of ratios $\Delta_{\left(g_{a},g_{b}\right)}/TR_{\left(g_{a},g_{b}\right)}^{I}$ computed ∀(*g*_*a*_,*g*_*b*_) corresponding to non-user-assigned $\Delta_{\left(g_{a},g_{b}\right)}$. Such a function is suitable for use in all situations in which the relationships between existing variables for which the user does not provide increments are to be maintained as far as is possible.

### Establishing the disease status

Once the coefficients of the MLM are fixed, the disease risk for each individual in a population can be established by substituting the coefficients associated with the carried genotype into Eq. (1) and then by evaluating the resulting logistic function forthe exposure level of the environmental disease factor. Finally, to assign the disease status to each individual, the disease risk is compared with a random number drawn from a uniform distribution.

## Availability and requirements

**Project name:** Gene-Environment iNteraction Simulator 2

**Project home page:**http://sourceforge.net/projects/gensim/

**Operating system(s):** Platform independent

**Programming language:** Python

**Other requirements:** SimuPop, OpenOpt, wxPython (optional)

**License:** GNU GPLv3

## Competing interests

The authors declare that they have no competing interests.

## Authors’ contributions

MP conceived the model and the extensions, and drafted the manuscript; GS conceived and developed the extensions, implemented the software and drafted the manuscript; RA conceived the model and the extensions and drafted the manuscript; SC and GM conceived the study, and participated in its design and coordination and helped to draft the manuscript. All authors have read and approved the final manuscript.

## Supplementary Material

Additional file 1 The GENS2 graphic user interface. Flowchart showing a typical way of using GENS2 through its graphical user interface. Portable Network Graphics (.png) image file. Click here for file

Additional file 2 Association test in the case of additive G×E. The population comprised 1,000 cases and 1,000 controls. Two DPLs (RR=1.6, W=0.5) in an additive G×E model (OR=1.2) with no epistatic interaction were present. The two DPLs are in two distinct genomic regions (Chr 8: 115,755,575-120,750,913 in yellow; Chr 10: 112,253,020-117,247,095 in cyan). In the upper panel, the Manhattan plot shows the significance of the association (−log_10_(p-value)) of each marker when tested individually (each dot represents a different marker). The red dashed line represents the significance threshold (0.05 after Bonferroni correction) and the green dashed lines mark the position of the DPLs. In the bottom panel, the *r*^2^ for each marker with the DPL in the same region is shown. Portable Network Graphics (.png) image file. Click here for file

Additional file 3 Association test in the case of modulative G×E. The population comprised 10,000 cases and 10,000 controls. Two DPLs (RR=1.6, W=0.5) in a modulative G×E model (OR=1.2) with no epistatic interaction were present. The two DPLs are in two distinct genomic regions (Chr 8: 115,755,575-120,750,913 in yellow; Chr 10: 112,253,020-117,247,095 in cyan). In the upper panel, the two Manhattan plots show the significance of the association (−log_10_(p-value)) of each marker when tested individually (each dot represents a different marker), using a multiplicative and an additive model in the logistic regression. The red dashed line represents the significance threshold (0.05 after Bonferroni correction) and the green dashed lines mark the position of DPLs. In the bottom panel, the *r*^2^ of each marker with the DPL on the same region is shown. Portable Network Graphics (.png) image file. Click here for file

Additional file 4 Expected and observed penetrance values plotted for each combined genotype and for different sample sizes. In each of the panels one of the possible combined genotypes is shown. The genotypes (1, 2, and 3) are ordered according to their predicted affect on the overall disease risk. The x-axes show the sample size and the y-axes show the risk. The green lines represent the expected risk, the blue lines show the median observed risk, and the red dashed lines indicate the minimum and maximum observed disease risk in 100 replicates. Portable Network Graphics (.png) image file. Click here for file
