# Supplementary figures and images for: Simulating gene-gene and gene-environment interactions in complex diseases: Gene-Environment iNteraction Simulator 2
Source: BMC Bioinformatics. 2012 Jun 14;13:132. doi: 10.1186/1471-2105-13-132 (PMC3538511; doi:10.1186/1471-2105-13-132)

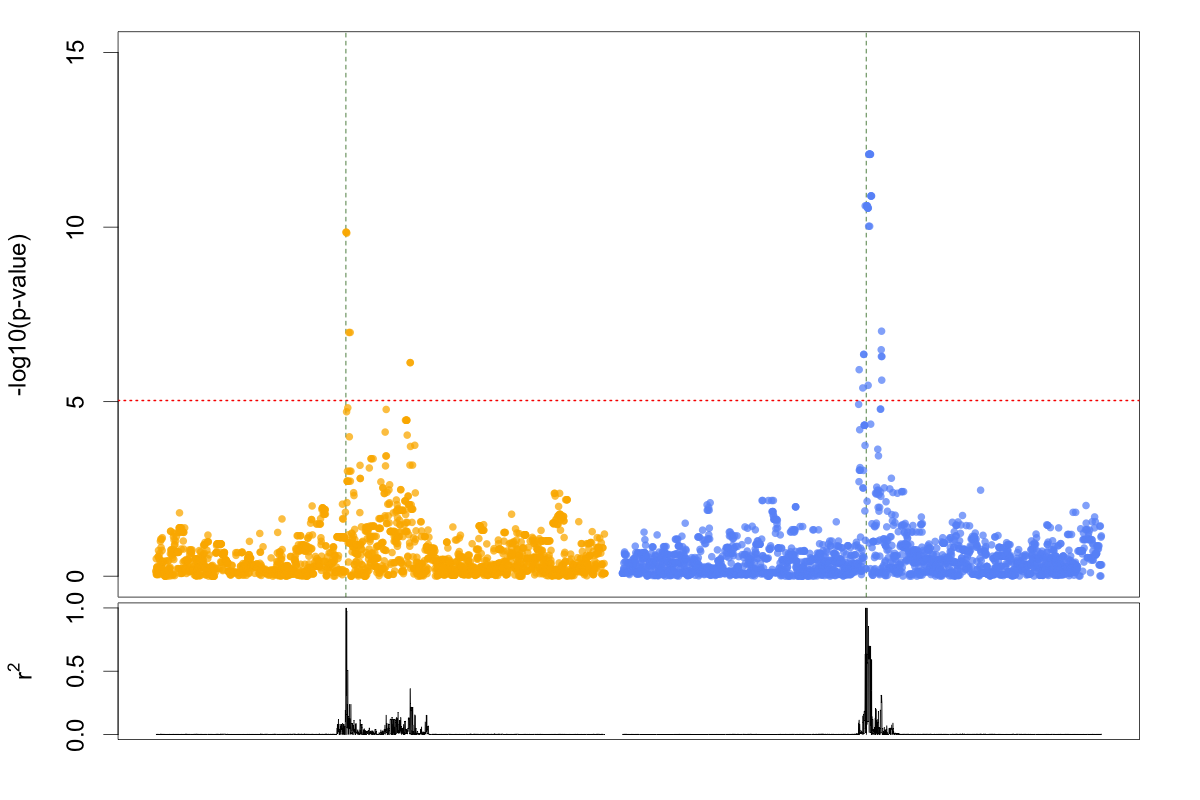

Supplement: Additional file 1 — The GENS2 graphic user interface. Flowchart showing a typical way of using GENS2 through its graphical user interface. Portable Network Graphics (.png) image file. [file 1471-2105-13-132-S1.png]

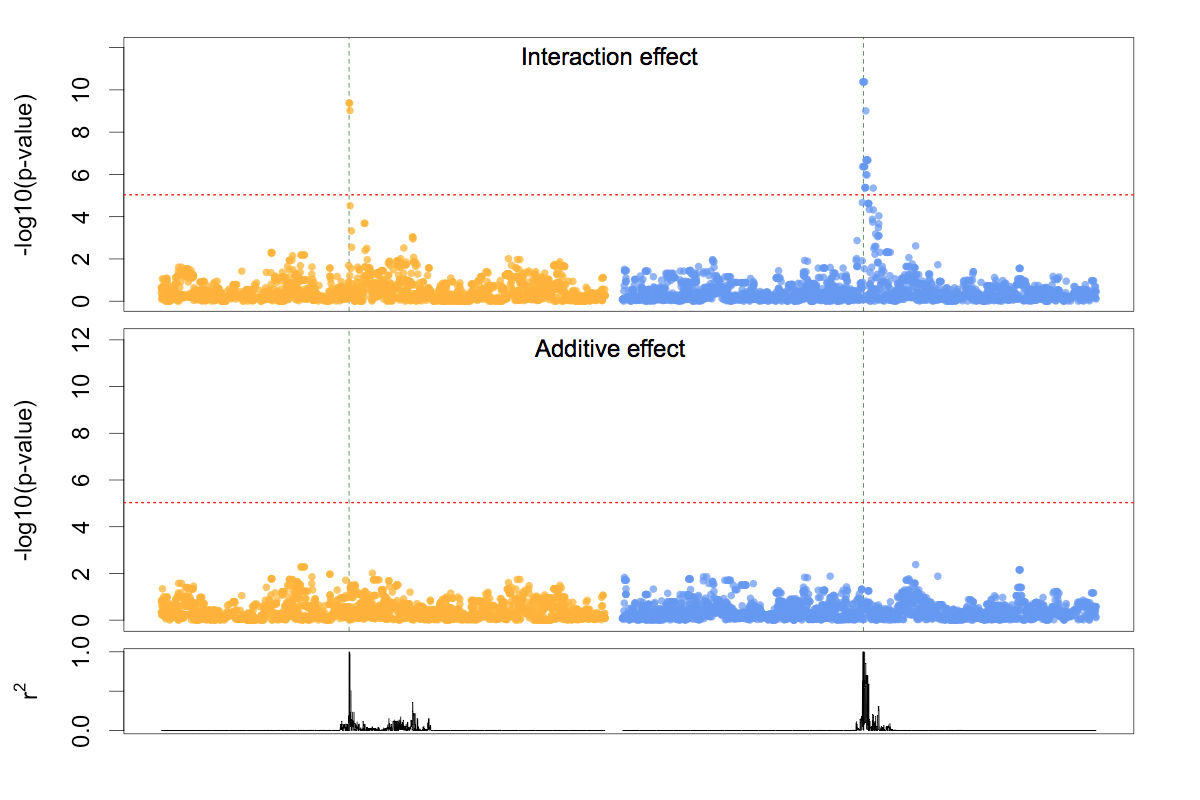

Supplement: Additional file 2 — Association test in the case of additive G×E. The population comprised 1,000 cases and 1,000 controls. Two DPLs (RR=1.6, W=0.5) in an additive G×E model (OR=1.2) with no epistatic interaction were present. The two DPLs are in two distinct genomic regions (Chr 8: 115,755,575-120,750,913 in yellow; Chr 10: 112,253,020-117,247,095 in cyan). In the upper panel, the Manhattan plot shows the significance of the association (−log10(p-value)) of each marker when tested individually (each dot represents a different marker). The red dashed line represents the significance threshold (0.05 after Bonferroni correction) and the green dashed lines mark the position of the DPLs. In the bottom panel, the r2 for each marker with the DPL in the same region is shown. Portable Network Graphics (.png) image file. [file 1471-2105-13-132-S2.png]

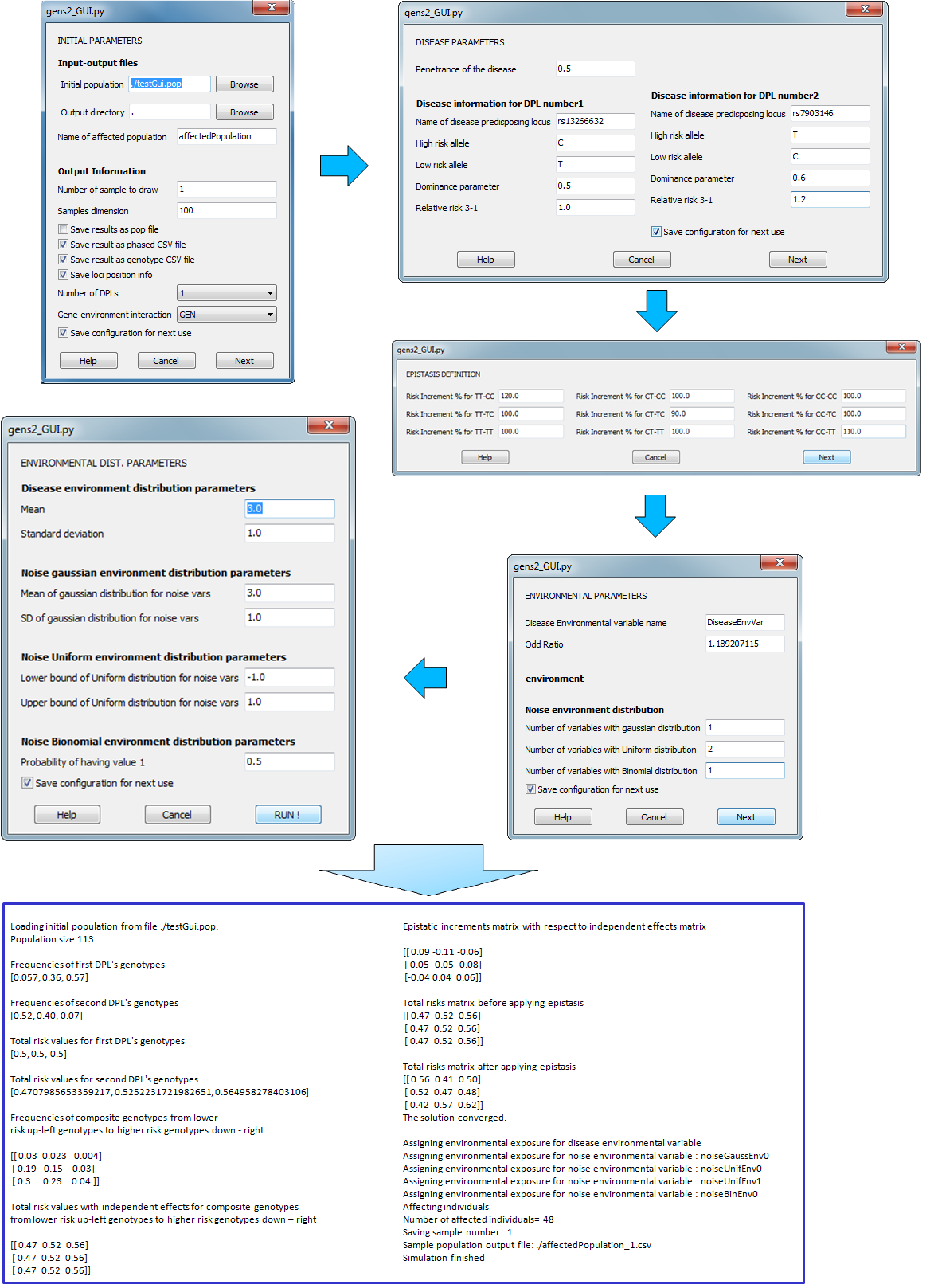

Supplement: Additional file 3 — Association test in the case of modulative G×E. The population comprised 10,000 cases and 10,000 controls. Two DPLs (RR=1.6, W=0.5) in a modulative G×E model (OR=1.2) with no epistatic interaction were present. The two DPLs are in two distinct genomic regions (Chr 8: 115,755,575-120,750,913 in yellow; Chr 10: 112,253,020-117,247,095 in cyan). In the upper panel, the two Manhattan plots show the significance of the association (−log10(p-value)) of each marker when tested individually (each dot represents a different marker), using a multiplicative and an additive model in the logistic regression. The red dashed line represents the significance threshold (0.05 after Bonferroni correction) and the green dashed lines mark the position of DPLs. In the bottom panel, the r2 of each marker with the DPL on the same region is shown. Portable Network Graphics (.png) image file. [file 1471-2105-13-132-S3.png]

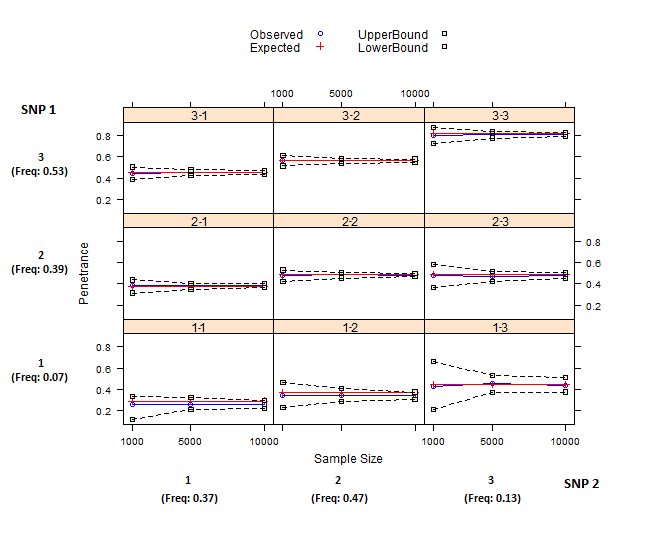

Supplement: Additional file 4 — Expected and observed penetrance values plotted for each combined genotype and for different sample sizes. In each of the panels one of the possible combined genotypes is shown. The genotypes (1, 2, and 3) are ordered according to their predicted affect on the overall disease risk. The x-axes show the sample size and the y-axes show the risk. The green lines represent the expected risk, the blue lines show the median observed risk, and the red dashed lines indicate the minimum and maximum observed disease risk in 100 replicates. Portable Network Graphics (.png) image file. [file 1471-2105-13-132-S4.png]
